# Electrochemical *N*-Acetyl-β-D-glucosaminidase Urinalysis: Toward Sensor Chip-Based Diagnostics of Kidney Malfunction

**DOI:** 10.3390/biom11101433

**Published:** 2021-09-30

**Authors:** Piyanuch Vibulcharoenkitja, Wipa Suginta, Albert Schulte

**Affiliations:** School of Biomolecular Science and Engineering (BSE), Vidyasirimedhi Institute of Science and Technology (VISTEC), 555/1 Payupnai, Wangchan, Rayong 21210, Thailand; piyanuch.v_s18@vistec.ac.th (P.V.); wipa.s@vistec.ac.th (W.S.)

**Keywords:** kidney, *N*-acetylglucosaminidase, disease biomarker, urine, urinalysis, voltammetry, diagnostics, nitrophenol, enzyme assay

## Abstract

*N*-Acetyl-β-D-glucosaminidase (GlcNAcase) is a valuable biomarker for kidney health, as an increased urinary level of the enzyme indicates cell damage within the renal tubular filtration system from acute or chronic organ injury or exposure to nephrotoxic compounds. Effective renal function is vital for physiological homeostasis, and early detection of acute or chronic renal malfunction is critically important for timely treatment decisions. Here, we introduce a novel option for electrochemical urinalysis of GlcNAcase, based on anodic differential pulse voltammetry at boron-doped diamond disk sensors of the oxidizable product 4-nitrophenol (4NP), which is released by the action of GlcNAcase on the synthetic substrate 4NP-*N*-acetyl-β-D-glucosaminide (GlcNAc-4NP), added to the test solution as a reporter molecule. The proposed voltammetric enzyme activity screen accurately distinguishes urine samples of normal, slightly elevated and critically high urinary GlcNAcase content without interference from other urinary constituents. Moreover, this practice has the potential to be adapted for use in a hand-held device for application in clinical laboratories by physicians or in personal home health care. Evidence is also presented for the effective management of the procedure with mass-producible screen-printed sensor chip platforms.

## 1. Introduction

The kidneys are major components of the urinary system; their functions include the elimination of waste and toxic species from the blood, regulation of blood volume and pressure, control of electrolyte and metabolite levels and the maintenance of blood pH [1]. Blood entering through paired renal arteries passes through the glomerulus/nephron network and after completion of decontamination it is returned to the circulatory system through the renal veins, while excess water and unwanted chemical species pass through the ureter as urine. Any reduction in the kidneys’ ability to regulate blood composition and pressure is a serious health threat, and if left untreated for long, leads to severe body degradation that may ultimately be fatal. In response to, for example, physical injuries with severe blood loss, sepsis or the destructive impact of nephrotic toxins, an abrupt, critical loss of function may result from an acute kidney injury [2,3]. On the other hand, long-term diabetes, continuous high blood pressure, cardiovascular problems, obesity, genetic vectors and/or smoking may cause the decline in renal capacity, which manifests as chronic kidney disease [4,5]. Obviously, analytical methods that can provide an early warning of deteriorating kidney function are useful diagnostic tools for clinicians and individuals from high-risk groups. The most widely used indicator of kidney function is the serum creatinine concentration, which is measured in clinical test centers to estimate the glomerular filtration rate, and thus the passage of blood through the kidneys [6,7,8]. However, a practical drawback of creatinine-based assessment of kidney health is the slow and non-specific manifestation of renal malfunction as detectable changes in the serum creatinine level; therefore, the onset and early stage of the degradation and thus the best time for commencing treatment may be missed [9,10,11,12]. To improve this situation, complementary urinary and serum biomarker assays with a better focus have been proposed for the detection and monitoring of acute [13,14,15,16] and chronic [17,18,19,20] renal disorders. Examples of suggested assay biomarkers include kidney injury molecule-1 (KIM-1), cystatin C, neutrophil gelatinase-associated lipocalin (NGAL) and *N*-Acetyl-β-D-glucosaminidase (GlcNAcase), to name just a few. Benchtop spectroscopic methodologies are usually employed to measure these biomarkers. Despite extensive work with various diagnostic and prognostic signaling molecules, there is no simple urinalysis procedure available that could be conveniently and efficiently applied by technical staff in clinical laboratories, doctors in general practice and individuals from high-risk groups at home, as a quick and reliable kidney health prescreen with diagnostic potential.

GlcNAcase is a 140-kDa lysosomal brush-border enzyme in proximal renal tubule epithelial cells that does not normally enter the urine at significant rates [21,22]. Thus, healthy urinary GlcNAcase levels are very low, while significant GlcNAcase elevation indicates renal tubule cell injury and is a critical sign of prevailing upstream renal problems. Following earlier reviews [21,22], a summary of the potential of urinary GlcNAcase testing [23] and topical reports on GlcNAcase electrochemical, electro-optical and optical analysis [24,25,26,27,28,29], we recently established a sensitive, real-time electrochemical (EC) assay for straightforward GlcNAcase activity determination. We used 4-methylumbelliferyl-N-acetyl-β-D-glucosaminide (GlcNAc-4MU) as a redox-labelled GlcNAcase substrate, with anodic oxidation of enzymically released 4-methylumbelliferone (4MU) at boron-doped diamond (BDD) disk working electrodes (WEs) for scalable amperometric signal generation [30]. Two purely electrochemical strategies that used a redox-labelled GlcNAcase substrate and detection by simple amperometry [24,30] had the potential for personal health care application but were not applied to human urine samples and were used simply as alternative assays of GlcNAcase activity in synthetic preparations. Tanaka et al. [25] developed a lab-on-a-compact disc platform with automated liquid handling and fluorescence detection for GlcNAcase measurements in artificial samples that simulated human urine. Ma et al. [28] determined GlcNAcase in human serum and urine using an optical assay that relied on the quenching of fluorescence of carbon nanodots by 4-nitrophenol released from 4-nitrophenyl-N-acetyl-β-D-glucosaminide (GlcNAc-4NP). Wang et al. [26,27] used electrochemiluminescence-based immuno-sensing to assess GlcNAcase levels in human serum. Finally, Kumar et al. [29] described a biosensor platform that quantified GlcNAcase in milk samples by reflectance spectrometry with a porous silicon Fabry−Peŕot interferometer. These analytical procedures performed well in GlcNAcase activity assays and are promising options for kidney biomarker urinalysis in well-equipped modern clinical laboratories. However, the complexity of their sensor platforms and their spectroscopic signal generation makes them unsuitable for personal urinary GlcNAcase monitoring with small hand-held devices that employ mass-fabricated, single-use sensor strips for detection.

Kidney health screening, whether with optical or electrochemical signaling, is a rather complex task. Sensor devices like the blood glucose meters available for diabetes patients or the immunosensor strips used in cancer biomarker diagnostics do not yet exist for kidney testing, apart from a commercial handheld meter for creatinine. This is a potentially useful tool; however, because of the limited data on blood or urinary creatinine, such as the delayed response to kidney deterioration, poor correlation of the variation in creatinine level with kidney problems and the known crosstalk with other diseases [19], additional complementary portable kidney screening devices would certainly be an asset for the health care system and for patients at risk. This is even more important with the growing number of kidney problem cases in an aging world population with increasing exposure to unhealthy diets and lifestyles. However, despite the urgent need, there is no report of a successful product for the health care market.

Using an adaptation of our previously established GlcNAcase electroanalysis [30], we describe here simple voltammetric detection of GlcNAcase as a biomarker of proximal tubule epithelial cell damage in the complex medium of urine samples. Procedural optimizations included the replacement of GlcNAc-4MU by GlcNAc-4NP, a three-fold cheaper redox-labeled GlcNAcase substrate, the use of a cheap and portable potentiostat instead of a bulky desktop model, and the use of differential pulse voltammetry (DPV) instead of amperometry for redox label electroanalysis to remove capacitive charging contributions to measured currents and improve signaling. The validation of this methodology involved model sample trials with GlcNAcase in sodium phosphate buffer solution and tests of actual urine with added GlcNAcase. Zero, low and high GlcNAcase levels were consistently identified for both sample types. We then demonstrated the feasibility of operation on mass-producible screen-printed electrode (SPE) platforms with a BDD WE disk, thus laying the foundation for an electrochemical urinary GlcNAcase strip test. The use of this test in an approved professional clinical trial on libraries of samples from patients with recognized kidney problems and from healthy control subjects, and the design and further realization of easy-to-use SPE-based GlcNAcase sensing strips in a portable device for personal kidney homecare testing will be the next issues addressed.

## 2. Materials and Methods

Standard chemicals for electrolyte preparation and 4-nitrophenol were obtained from S.M. Chemical Supplies Co., Ltd. (Bangkok, Thailand) as analytical grade Sigma-Aldrich Corporation (St. Louis, Missouri, US) reagents. The chosen biomarker for kidney defects, *N*-Acetyl-β-D-glucosaminidase (EC 3.2.1.52), was purchased as a purified enzyme preparation from *Canavalia ensiformis* (Jack bean), from the same suppliers. Monobasic (NaH_2_PO_4_) and dibasic (Na_2_HPO_4_) sodium phosphate for sodium phosphate buffer solution preparation were from Italmar (Thailand) Co., Ltd., (Bangkok, Thailand). Ultrapure de-ionized water was used for all solutions.

All electroanalysis was conducted with a portable PalmSens4 mini-potentiostat (Palmsens BV, GA Houten, The Netherlands). For voltammetry execution, the device was controlled by its own software, PS Trace, Edition 5.8. Collected data were analyzed with the PS trace software and graphs visualizing the obtained results were constructed by using GraphPad Prism 8 and Microsoft Excel and PowerPoint 2019 software.

Voltammetry was carried out in a three-electrode electrochemical cell with a 3-mm-diameter BDD disk working electrode (Windsor Scientific Ltd., Slough Berkshire, UK), a platinum (Pt) sheet counter electrode (Metrohm Siam Ltd., Bangkok, Thailand) and an Ag/AgCl (3 M KCl) reference electrode immersed in 3 mL of electrolyte in a small glass beaker. Test solutions were 0.1 M PBS pH 7.0 with or without 4NP or urine sample supplementation. Before measurements, the surface of a used BDD disk was regenerated by polishing with alumina powder with a particle size of 0.4 μm to optimize 4NP detection. The procedure involved a thick suspension of the alumina in water and a handheld dental drill. A tiny drop of the alumina slurry was placed on the BDD disk and a few minutes of gentle polishing with the soft rubber-covered tip of the fast-rotating bit was used for cleaning.

Commercial screen-printed electrodes with integrated 3.6-mm-diameter BDD disk sensors (Metrohm Siam Ltd., Bangkok, Thailand) were tested as alternative sensor strips for urinary GlcNAcase assessments. They were immersed in 5 mL of 0.1 M PBS pH 7.0 with the addition of urine or urine/GlcNAcase. 4NP voltammetry used a three-electrode configuration, but to allow better comparison with standard BDD electrode voltammetry, it was performed with the Pt-sheet and the Ag/AgCl (3 M KCl) used in standard trials serving as external equivalents, rather than the built-in counter- and reference-electrodes of the SPE platform.

Solutions of 0.1 M PBS, pH 7.0 with additions of 5, 20 and 100 U/L GlcNAcase served as model samples. Urine for real sample tests was from a healthy investigator in this study who approved its use for the purpose of electrochemical inspection. Collections from other individuals were not involved and formal institutional study authorization, required for medical trials on pools of body fluid or tissue from patients, was therefore unnecessary.

## 3. Results

**Scheme 1:**
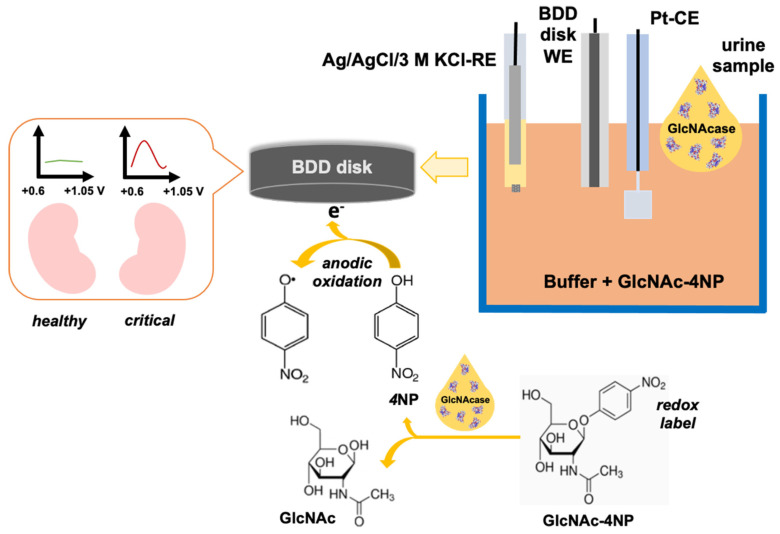
The proposed electrochemical assay for the detection of kidney status biomarker, *N*-Acetyl-β-D-glucosaminidase (GlcNAcase) in the urine of patients with suspected renal damage. Key components of the method are the three-electrode electrochemical cell arrangement with a boron-doped diamond disk (BDD) working-electrode, a Pt plate counter-electrode and an Ag/AgCl/3 M KCl reference electrode for voltammetric 4NP detection, a handheld portable potentiostat for voltammetry, and finally 4NP, a redox-labeled GlcNAcase product that is added to the buffered supporting electrolyte. GlcNAcase, from a urine sample with low (normal) or high (acute) levels, liberates the 4NP redox label from its sugar substituent by glycosidic bond cleavage. Released 4NP is sensed at the BDD electrode by electro-oxidation in a voltammetry mode; low or large voltammetric peak currents are diagnostic indicators of healthy or critical kidney state.

The principle of the proposed voltammetric urinary GlcNAcase assay is shown in Scheme 1. Kidney damage results in an increase in the urinary concentration of GlcNAcase, which in our assay catalyzes the release of 4-NP from the synthetic substrate, and this is detected as an electrochemical signal.

A three-electrode electrochemical cell is set in a 5 mL glass beaker with a BDD disk, Pt plate and an Ag/AgCl (3M KCl) system, referred to as WE, CE and RE, respectively, operated in 0.1 M PBS buffer. For GlcNAcase measurements, the buffered electrolyte was supplemented with GlcNAc-4NP, which is a substrate of the biomarker GlcNAcase and releases the redox-active, 4NP, the signaling molecule in the assay. Earlier work reported pH 7.0 and 37 °C to be optimal for GlcNAcase assays [30,31]. Here the buffer was at pH 7.0, but the assay was conducted at room temperature (25 °C) since the ultimate application would be for personal kidney testing at home, where measurements at constant elevated temperature would not be practicable. The time allowed for GlcNAcase, either as a supplement of PBS model solutions or as a biomarker in urine samples, to act on GlcNAc-4NP in the assay buffer, was varied between 5 and 60 min before the final assessment of liberated 4-NP was carried out by DPV analysis.

Also important for a successful assay was a suitable choice of the WE and its protection against electrode fouling, which adversely affects the electrochemical detection of 4-NP, and phenols in general, and is related to electro-polymerization following radical formation in the anodic oxidation of phenolic analytes. Unwanted polymer film formation on the sensor surface could affect electron transfer, and thus lead to a decrease in the quality of the 4-NP measurements. A recent review provides an overview of the strategies that are available to protect electrochemical phenol sensing against electrode activity degradation [32]. For our analysis, a reusable conductive BDD disk was chosen as the sensor because these disks suffer much less than other common electrode materials (e.g., glassy carbon, platinum or gold) from phenol-related losses of performance quality [33,34,35]. To further ensure that the 4-NP measurements were reliably reproduced between analytical sample runs, we additionally polished the electroactive disk of the BDD electrode between individual measurements to remove any polymeric residue that might have formed and reset sensors to the same quality for every recording. That this strategy worked satisfactorily was confirmed through the execution of triplicate DPV test runs on 500 μM solutions of 4-NP in PBS buffer: the three recorded DPV plots could be overlaid virtually perfectly, with peak currents of 33.3 μA and a small standard deviation of 0.04 μA (not shown).

**Figure 1:**
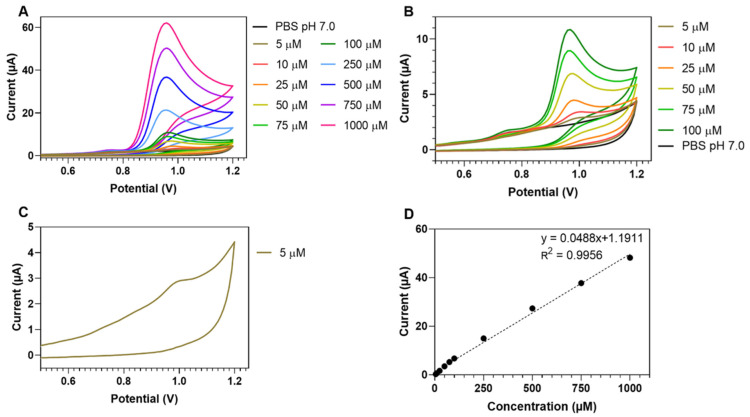
Cyclic voltammograms (CVs) from a 3-mm-diameter BDD disk electrode in 0.1 M PBS pH 7.0 and in 0.1 M PBS pH 7.0 with 5–1000 µM 4NP (**A**). Zoomed CV display for 5–100 µM 4NP (**B**) and 5 µM 4NP (**C**). Calibration plot (mean 4NP CV peak current values from threefold repeats vs. actual 4NP level) for 4NP cyclic voltammetry at BDD disk working electrodes (**D**). Note: Error bars in (**D**) are smaller than the symbols of the data points. CV scan speed was 0.05 V s^−1^.

**Figure 2:**
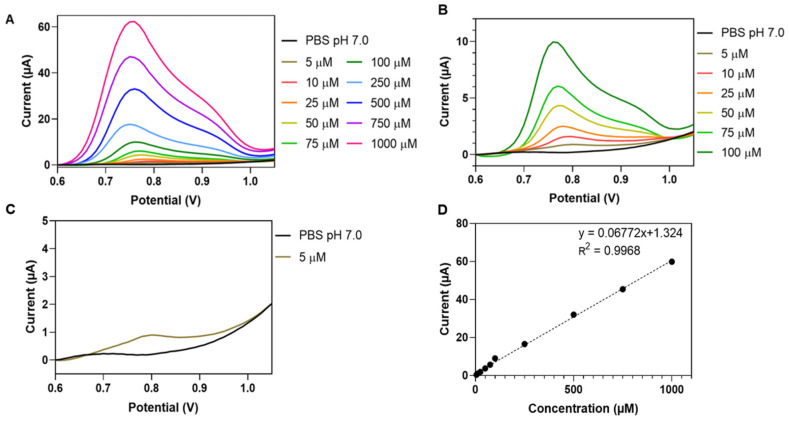
Differential pulse voltammograms (DPVs) from a 3-mm-diameter BDD electrode in 0.1 M PBS pH 7.0 and 0.1 M PBS with 5–1000 µM 4NP (**A**). Zoomed DPV display for 5–100 µM 4NP (**B**) and 5 µM 4NP (**C**). Calibration plot (mean 4NP DPV peak current values from triplicate repetitions vs. actual 4NP level) for 4NP differential pulse voltammetry at BDD disk working electrodes (**D**). Note: Error bars in (**D**) are smaller than the symbols of the data points. DPV scan speed was 0.05 V s-1 while the pulse amplitude, step and width were 0.2 V, 0.01 V and 0.02 s.

When the urine of a donor contains GlcNAcase because of renal damage and consequent leakage into the tubular lumen, addition of the sample to the electrolyte will cause enhanced release of 4NP, compared to the urine of a healthy person. The three-electrode arrangement transforms enzymic generation of free 4NP into a quantifiable electrical signal that can be calibrated to allow calculation of the GlcNAcase concentration in the urine sample. The responsiveness of BDD disk electrodes to the redox-active signaling molecule in the assay was tested by cyclic and pulse voltammetry in PBS, pH 7.0 with increasing concentrations of 4NP. Figure 1 and Figure 2 are cyclic (CV) and differential pulse (DPV) voltammograms that were obtained with the defined three-electrode set operating in solutions of 0–1000 µM 4NP in PBS. The CVs contained tiny current elevations at the base of the main peaks from the anodic oxidation of 4-NP. Zooms of the “foot” signals for the traces in Figure 1A,B (not shown) showed that they were also present with buffer alone, at the same potential and with the same magnitude for all 4-NP concentrations. Obviously, this tiny, reproducible trace feature is unrelated to the target analyte, 4-NP, but originates either from a trace contaminant in the PBS buffer or, more likely, it is a characteristic of the polished BDD disk working electrode, which undergoes an anodic redox reaction, such as surface carbon atom oxidation at that potential. However, since the artefacts are minor compared to the height of the analytically relevant 4-NP peak, their presence did not detract from the performance of our voltammetric GlcNAcase assay as the peaks of DPV recordings, which are the basis of our urinary assay, were free from the artefact. The slight DPV peak asymmetry and shoulder manifestation are likely a sign that oligomerization of radical intermediates of the primary 4-NP electrode interaction, and to a minor extent oligomer electrooxidation, took place at the anodically polarized BDD disk, but at slightly higher potentials than are needed for electron transfer from monomeric 4-NP. Extraction of anodic CV and DPV peak currents from triplicate repetitions of the voltammetric measurements and plotting the mean values against 4NP concentrations produced regression graphs with small data point standard deviations (<2%) and good linearity (R^2^ = 0.9959 (CV) and R^2^ = 0.9968 (DPV)). Carefully polished BDD disk electrodes achieved reliable voltammetric 4NP detection, with about 5 µM being the lowest concentration to produce analyzable anodic CV (Figure 1C) and DPV (Figure 2C) current peaks, which defined the practical limit of the two methodologies. Since the current peaks recorded in the differential pulse voltammetry mode were largely free of contributions from electrode capacitance charging currents, all further electrochemical 4NP tests used this practice. As a first step, positive 4NP redox label voltammetry was acquired by measurements of the target species in PBS solutions.

**Figure 3:**
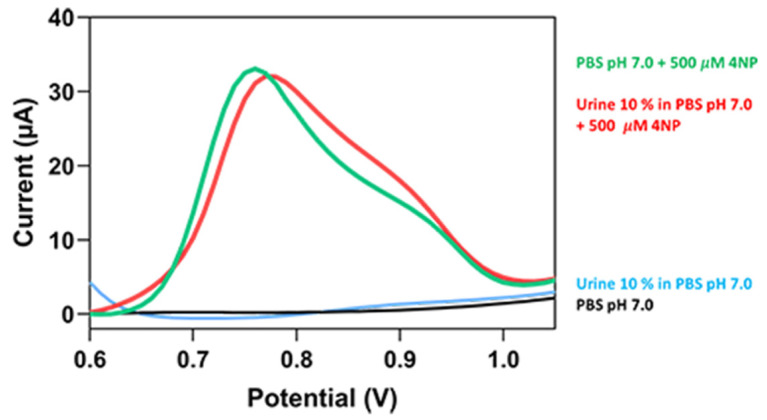
Differential pulse voltammograms (DPVs) from a 3-mm-diameter BDD disk electrode in 0.1 M PBS pH 7.0 containing 0 (black trace) and 500 µM (green trace) 4NP. Light blue and red traces are DPVs obtained through the operation of a BDD sensor in urine diluted 10-fold in 0.1 M PBS pH 7.0 without and with the addition of 500 µM 4NP, respectively. Parameters for DPV acquisition were as listed in Figure 2.

Bearing in mind the application for electrochemical urinalysis, we inspected the DPV response of BDD disks in buffers containing 4NP and a urine addition that might cause signal interference. Figure 3 shows the results of the interference trials that were required to confirm the selectivity of the assay in measurements involving real urine. For scans across the potential range used for 4NP DPV acquisition, the BDD disk currents were virtually zero with pure PBS, pH 7.0 and with urine diluted 10-fold in buffer. The addition of 500 µM 4NP to urine-free and urine-containing PBS produced anodic current waves equivalent to those in the initial triplicate 4NP DPV calibration trials for both electrolyte conditions, indicating concentration-dependent Faradaic 4NP oxidation. This was demonstrated by the pronounced similarity of the DPVs in Figure 2 and Figure 3 and the observation that peak shapes and peak magnitudes for the identical analyte adjustment matched adequately. Although urine is a complex chemical mixture, at 10-fold dilution it apparently introduced no component with a formal reduction potential and concentration that could produce an anodic oxidation current high enough to interfere with DPV detection of 4NP, the signaling molecule in the assay. Undiluted urine is more critical because of significant residual background currents in the region of the 4-NP detection potential and would require the development of special interference and/or detection tactics.

Lysosomal GlcNAcase in circulating blood is too large to pass through the filtration system of the renal glomeruli into the urine; however, it might enter urine in tiny amounts by, for instance, lysosomal exocytosis. The urine of healthy people with normal kidney function is thus not entirely free of GlcNAcase, but its level is low with 5–10 U/L having been reported for control populations in clinical studies [36,37]. The elevated and critical levels are 20 and 100 U/L or higher, respectively. Thus, the model samples for the initial tests of our electrochemical GlcNAcase assay were 0.1 M PBS, pH 7.0 solutions with “healthy” (5 U/L), “concerning” (20 U/L) and “critical” (100 U/L) additions of GlcNAcase.

**Figure 4:**
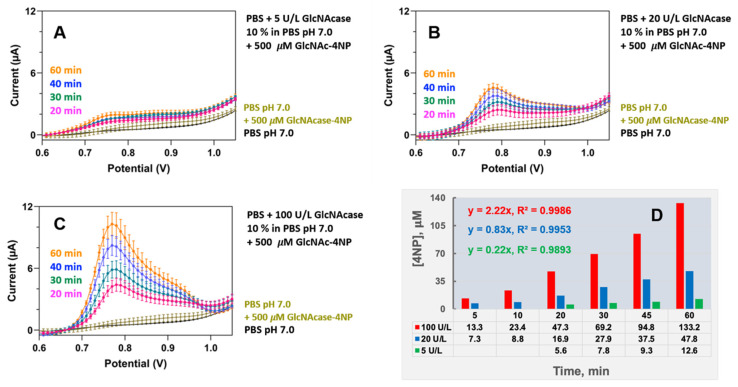
Assessment of low (5 U/L, (**A**)), medium (20 U/L, (**B**)) and high (100 U/L, (**C**)) levels of the kidney status biomarker N-acetylglucosaminidase (GlcNAcase) in 0.1 M PBS, pH 7.0 model samples. A total of 300 µL of the simulated urine samples was added to 2700 µL of 0.1 M PBS, pH 7.0 containing 0.5 mM GlcNAc-4NP. 4NP differential pulse voltammograms (DPVs), recorded 20, 30, 45 and 60 min after initiation of the trial, visualized 4NP release through the concentration-dependent action of GlcNAcase on the labelled substrate with progressively increasing anodic peak currents. (**D**) Bar chart plot of anodic 4NP peak current magnitudes, i_p_, as functions of catalytic time. Insets in (**D**) are the slopes of regression lines through plots of the i_p_ values as listed below the x-axes vs. corresponding reaction time. The average percentage standard deviation for the y-axes data point values of the triplicate trial, not specifically visualized here as error bars, was (11 ± 5)%. Parameters for DPV acquisition were as listed in Figure 2.

For the 5 U/L GlcNAcase sample present in the electrochemical cell at 0.5 U/L after dilution, a minimum of 20 min was required to raise the concentration of 4NP produced by cleavage of GlcNAc-4NP to achieve significant elevation in the anodic WE current above the background, and even after a full hour of reaction, the observed DPV peaks were faint (Figure 4A). At the same dilution the 20 and 100 U/L GlcNAcase samples both produced clear, progressively increasing anodic peak currents (Figure 4B,C). Bar chart plots of the anodic 4NP peak current magnitudes, i_p_, as a function of the GlcNAcase concentration and trial time, t, show the marked difference between the voltammetry of low, medium and high GlcNAcase-level test solutions (Figure 4D). The slopes of regression lines through the data points in plots of i_p_ vs. corresponding reaction time (not shown), which are the rates of GlcNAcase-induced growth in 4NP concentration for a particular trial condition, were 0.22, 0.83 and 2.22 µM/min for 5, 20 and 100 U/L GlcNAcase, respectively (Figure 4D, inset). The methodology was applied in the following trials to measure the GlcNAcase content of urine containing common physiological metabolites.

**Figure 5:**
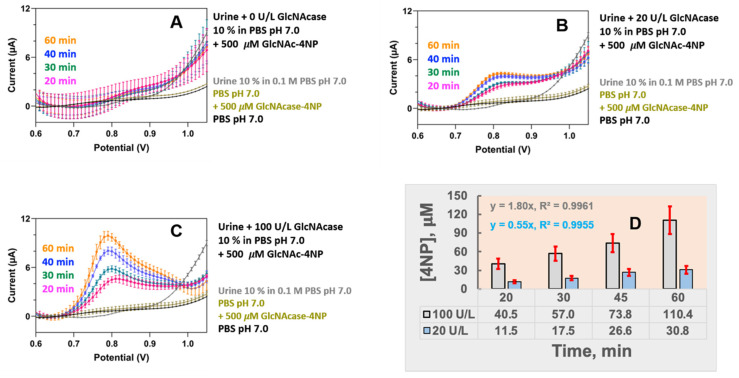
Assessment of model samples in 0.1 M PBS, pH 7.0: unsupplemented urine (**A**), urine with a medium (20 U/L, (**B)**) and urine with a high (100 U/L, (**C**)) concentration of the biomarker GlcNAcase. A total of 300 µL of the urine samples was added to 2700 µL of 0.1 M PBS, pH 7.0 containing 0.5 mM GlcNAc-4NP. 4NP differential pulse voltammograms (DPVs), recorded 20, 30, 45 and 60 min after trial start, visualized 4NP release through the action of GlcNAcase on the substrate as progressively increasing anodic peak currents. (**D**) Bar chart plot of anodic 4NP peak current magnitudes, i_p_, as a function of catalytic time GlcNAc-4NP. Insets are the slopes of regression lines through plots of the i_p_ values as listed below the x-axes vs. corresponding reaction time. The width of the error bars of all data points is ±20%, which was the observed worst-case scenario throughout all individual measurements of the triplicate trial with actual urine; average percentage standard deviation was (10 ± 6)%. Parameters for DPV acquisition were as listed in Figure 2.

Urine from hospital patients with kidney problems, especially those with confirmed renal tubular damage and associated elevated urinary GlcNAcase levels, was not available in the laboratory phase of assay development during this study. Further measurements were therefore made using urine from a healthy, unmedicated donor. Samples were tested unmodified or with the addition of 20 and 100 U/L GlcNAcase to mimic a concerning or severely critical state of the kidneys. Figure 5 is a representative display of the three sets of DPVs that were acquired for unmodified and GlcNAcase-supplemented urine samples 20, 30, 45 and 60 min after their 10x dilution into 0.1 M PBS, pH 7.0. For simple urine addition to measuring buffer with 0.5 mM GlcNAc-4NP no well-defined elevation of the anodic WE current over the urine background was observed in the four timetabled DPV recordings (Figure 5A). Apparently, the urine lacked electrochemically detectable levels of metabolic interferents and as expected for a healthy person’s urine, its content of GlcNAcase as an enzymic biomarker of kidney problems was also insignificant. However, 10-fold diluted urine samples with 20 or 100 U/L GlcNAcase added to the electrolyte with 0.5 mM GlcNAc-4NP increased the 4NP concentration enough to produce progressively increasing anodic current peaks (Figure 5B,C). As with the entirely artificial PBS/GlcNAcase samples, i_p_/t bar chart plots and a comparison of the slopes of the linear regressions through the data points in plots of i_p_ vs. corresponding reaction time showed a three- to four-fold difference between the voltammetry fingerprints of urine samples that simulated minor or major deterioration of kidney function (Figure 5D) while unmodified urine was voltammetrically silent.

**Figure 6:**
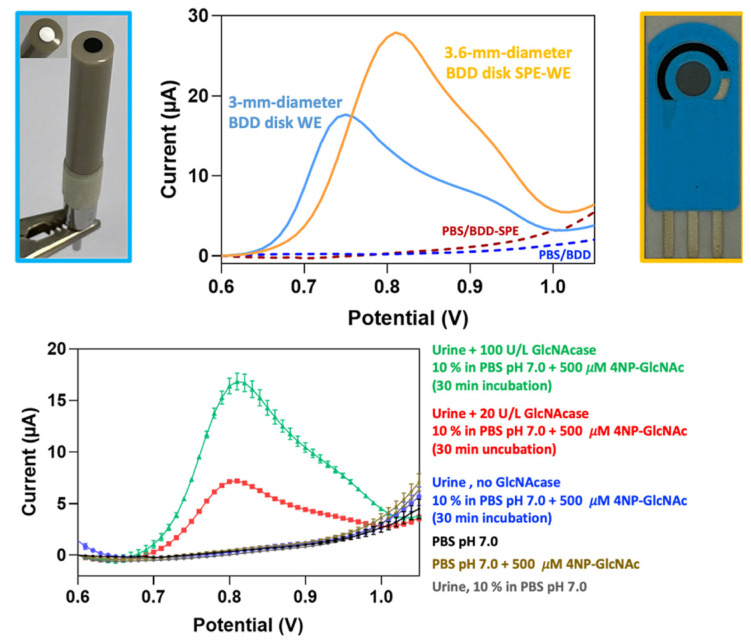
Application of screen-printed boron-doped diamond disk electrodes (BDD SPEs) to urinary N-acetylglucosaminidase (GlcNAcase) voltammetry. Comparison of the DPVs of a standard rod-type BDD disk electrode in 0.1 M PBS, pH 7.0 (top, dark blue trace) and in 0.1 M PBS, pH 7.0 supplemented with 500 µM 4NP (top, light blue trace) with the DPVs of a BDD SPE in 0.1 M PBS, pH 7.0 (top, red trace) and in 0.1 M PBS, pH 7.0 supplemented with 500 µM 4NP (top, orange trace). DPV assessment of unmodified urine (bottom, blue trace) and urine with medium (20 U/L, bottom, red trace) and high (100 U/L, bottom, green trace) added concentrations of GlcNAcase. DPVs were acquired in 0.1 M PBS, pH 7.0 with 0.5 mM GlcNAc-4NP 30 min after the addition of 300 µL of a particular urine sample to 2700 µL of buffered electrolyte and visualized 4NP release through the concentration-dependent action of GlcNAcase on the labelled substrate with progressively increasing anodic peak currents. The black and olive-colored traces refer to DPVs in 0.1 M PBS, pH 7.0 and in 0.1 M PBS, pH 7.0 with 0.5 mM GlcNAc-4NP but no GlcNAcase, respectively. Parameters for DPV acquisition were as listed in Figure 2.

The suitability of urinary GlcNAcase electroanalysis for routine decentralized clinical and personal kidney testing with a portable readout device was finally verified though the conversion of the assay from use with the standard rod-type BDD disc WE to operation on mass-producible planar BDD-SPE sensor platforms. With simple PBS pH 7.0 as the supporting electrolyte, both electrode types offered a featureless and virtually zero-current baseline DPV response (Figure 6, top, red and blue traces). The 20% larger disk diameter and some surface roughness in the tested screen-printed BDD SPEs produced DPV peak currents about 1.6-fold higher than for the BDD disk (Figure 6, top orange and light blue traces) at the same 4NP concentration. On the other hand, an anodic shift of about 60 mV in the BDD SPE peak potential was a sign of poorer electron transfer kinetics in the thick-film BDD SPE surface, as compared to the redox behavior of the smooth polycrystalline BDD layout. However, this effect did not obstruct practical 4NP voltammetry, and as demonstrated in the top and bottom parts of Figure 6, the performance of the BDD-SPE module was acceptable for urinary GlcNAcase electroanalysis. A 30 min incubation of urine samples in GlcNAc-4NP-containing test buffer reliably produced a clear distinction between the 4NP DPV peak current traces of normal, slightly elevated and critically high GlcNAcase levels in the model samples. Thus, the proposed assay strategy is able to deliver easy electrochemical signaling of abnormal GlcNAcase entry into urine and to provide a binary “Yes”/”No” report on renal tubular damage.

## 4. Conclusions

Thoroughly polished BDD disk electrodes were used in this study as voltammetric probes for 4NP detection down to the low micromolar level. In the differential pulse voltammetry mode in solutions that contained GlcNAcase together with a 4NP-labeled substrate, GlcNAc-4NP, the conductive diamond sensors reliably monitored GlcNAc-4NP hydrolysis and the resultant release of 4NP as gradually increasing magnitudes of consecutively acquired anodic DPV peaks. Following reports that the GlcNAcase content of urine is an effective biomarker for kidney degradation, and ultimately, organ failure due to cell damage in the renal filtration system, advantage was taken of the BDD/DPV-based enzyme activity assay for the completion of technically undemanding electrochemical GlcNAcase urinalysis. The strategy correctly identified and classified the manifestation of average healthy, moderately raised or seriously excessive GlcNAcase concentrations in urine samples. Furthermore, evidence was provided that the proposed voltammetric enzyme assay was successful in urinary GlcNAcase tests using mass-producible screen-printed BBD disk sensor platforms. Obviously, the proposed practice has the potential, with a suitably adapted design and with handheld electronic data acquisition and display devices, to ultimately become the first advanced sensor chip-based tool for rapid kidney function checks in clinical laboratories, physicians’ offices and at home, for convenient and straightforward personal urine checks.

## Data Availability

Data is contained within the article. Original traces of the voltammetry trials are available on request from the corresponding author.
